# A measurement-driven graph learning framework for Wideband oscillation localization in power systems

**DOI:** 10.1371/journal.pone.0355190

**Published:** 2026-08-04

**Authors:** Zhilin Dong, Haoqing Xiong, Jun Yang, Rongqiang Su, Qianfeng Shen, Shaohua Zhang, Yixin Chang, Jiaxu Chen, Xuejin Qian

**Affiliations:** 1 CHINA EPPEI Smart Energy Co., Ltd., Beijing, China; 2 China Electric Power Planning and Engineering Institute, Beijing, China; Commonwealth Scientific and Industrial Research Organisation, AUSTRALIA

## Abstract

Wideband oscillations in converter-dominated power systems exhibit complex multi-band characteristics, strong nonstationarity, and weak spatial coherence, posing significant challenges to conventional oscillation source localization methods that are primarily designed for narrowband low-frequency scenarios. In particular, medium- and high-frequency components tend to be highly localized and rapidly attenuated, making global-consistency-based approaches less effective. To address these challenges, this paper proposes a novel spatiotemporal graph learning framework, termed LCGS-Net (Local-Contrast Global-Smooth Network), for wideband oscillation source localization. The proposed method introduces a dual-branch representation mechanism that explicitly disentangles globally smooth propagation patterns from locally contrastive high-frequency perturbations. In addition, a hierarchical channel interaction module is developed to capture the coupling relationships among multi-channel measurements, enabling more expressive feature representations. An adaptive fusion strategy is further employed to dynamically balance local and global information. Simulation studies on IEEE benchmark systems demonstrate that the proposed method outperforms several representative temporal and graph-enhanced baseline models. In particular, LCGS-Net shows improved performance under high-frequency oscillation scenarios, where conventional smoothing-oriented graph aggregation methods may suppress localized source-related features. Additional experiments on the IEEE 39-bus system provide an initial validation of the scalability of the proposed framework. The results indicate that LCGS-Net can effectively capture local contrastive spatial characteristics of wideband oscillations in benchmark systems, while further validation on realistic large-scale converter-dominated grids remains necessary.

## 1 Introduction

With the increasing penetration of renewable energy sources, global power systems are undergoing a profound transition toward low-carbon and sustainable operation [1,2]. This transformation is primarily driven by the large-scale integration of power-electronic-interfaced generation, which is progressively replacing conventional synchronous machines and reshaping the fundamental dynamic characteristics of power systems [3,4]. To mitigate the decline in system inertia and maintain stable operation, grid-forming control strategies, such as virtual synchronous generators (VSGs), have been widely adopted to emulate the inertial and damping responses of synchronous machines [5,6]. However, unlike traditional generators, power electronic converters are governed by fast control loops and complex inner–outer control interactions, which significantly alter system dynamics [7,8]. As a result, modern power systems exhibit increasingly complex oscillatory behaviors across multiple time scales, posing new challenges for system stability analysis and monitoring [2,9].

More broadly, the modeling and analysis of complex dynamical systems with coupled transport processes, memory effects, and nonstationary responses have also been investigated in other engineering fields. For example, fractional-order models have been applied to magnetohydrodynamic and second-grade fluid systems to characterize memory-dependent dynamic responses and heat/mass transfer processes [10–13]. In addition, data-driven techniques such as artificial neural networks have been introduced to approximate nonlinear thermal-fluid dynamics under fractional operators [14]. Although these studies focus on fluid and heat-transfer systems rather than power-system oscillations, they provide useful evidence that complex nonstationary dynamics often require advanced mathematical modeling and data-driven representation learning.

Building upon the increasingly complex multi-timescale oscillatory behaviors observed in modern power systems, oscillation source localization has become a critical task for ensuring system stability and enabling effective mitigation actions [9]. In conventional power systems, where oscillations are typically concentrated in low-frequency or sub-/super-synchronous ranges, a variety of localization methods have been developed based on both model-driven and measurement-driven paradigms [15].

From a model-driven perspective, modal analysis and impedance-based approaches have been widely employed to infer oscillation sources by analyzing system eigenstructures or frequency-domain characteristics [16]. These methods offer strong physical interpretability and can effectively identify dominant oscillation modes and their associated sources when accurate system models are available [17].

From a measurement-driven perspective, energy-based and signal-correlation-based methods have been extensively investigated. Among these, dissipating energy flow (DEF) and its variants have emerged as representative techniques for locating sustained oscillation sources by analyzing the direction of oscillatory energy propagation [18]. In addition, mode-shape-based methods and transfer characteristic analysis approaches have also been proposed to estimate the spatial distribution of oscillation sources using wide-area measurements [19]. These methods have demonstrated satisfactory performance in synchronous-machine-dominated systems, particularly for forced oscillations and poorly damped low-frequency oscillations.

In recent years, data-driven and deep-learning-based methods have been increasingly introduced into oscillation source localization [20]. Unlike conventional approaches that rely on explicit physical models or predefined propagation indicators, these methods aim to learn discriminative spatiotemporal features directly from measurement data. Existing studies have explored several representative directions, including data-driven localization using synchrophasor measurements and inferred impulse responses, graph-based learning frameworks that incorporate network topology via temporal graph convolution, and knowledge-informed deep learning approaches that integrate physical priors such as dissipating energy flow [21]. Moreover, hybrid architectures that combine graph convolution, recurrent networks, and attention mechanisms have been proposed to capture complex spatiotemporal dependencies in wide-area measurements [22]. Recent studies have further introduced graph-enhanced variational representation learning and Transformer-based graph attention mechanisms to improve localization robustness and long-range spatiotemporal modeling capability under complex oscillation scenarios [23,24]. These developments highlight the strong potential of data-driven localization methods for handling nonlinear and complex dynamics in converter-dominated systems [25].

However, with the widespread deployment of power electronic converters and advanced control strategies, such as grid-forming and grid-following controls, new types of oscillations have emerged that span a much broader frequency spectrum [26]. These wideband oscillations are often induced by interactions among multiple control loops, including inner current loops, outer voltage or power control loops, and phase-locked loops, resulting in strongly coupled dynamics across different frequency bands [27]. Consequently, oscillatory components may simultaneously appear in low-frequency, sub-synchronous, and medium- to high-frequency ranges, exhibiting complex multi-band characteristics.

This evolution fundamentally challenges the applicability of conventional oscillation analysis and localization methods, which are predominantly designed for narrowband low-frequency oscillations. In traditional scenarios, oscillations are typically dominated by a small number of well-separated modes, characterized by relatively coherent spatial patterns and stable propagation characteristics. Under such conditions, model-driven approaches, energy-based methods, and even data-driven techniques can effectively identify oscillation sources by exploiting well-defined modal structures or clear energy flow directions.

In contrast, wideband oscillations—particularly those in the medium- and high-frequency ranges—exhibit markedly different characteristics [28–30]. First, multiple oscillatory components may coexist and overlap across frequency bands, resulting in strong modal coupling and making it difficult to isolate individual modes. Second, the fast dynamics introduced by converter control loops lead to highly nonstationary behaviors, thereby violating the quasi-stationary assumptions commonly adopted in traditional methods. Third, the propagation of high-frequency components is strongly influenced by network impedance characteristics, leading to rapid attenuation and spatial localization of oscillation energy. Collectively, these factors undermine the effectiveness of conventional localization indicators, such as coherent mode shapes or consistent energy flow patterns.

As a result, although recent studies have highlighted the emergence of wideband oscillations spanning from several hertz to several kilohertz, oscillation source localization in wideband scenarios—particularly for medium- and high-frequency oscillations—remains largely underexplored. This limitation becomes even more pronounced in converter-dominated systems, where oscillatory behaviors are inherently broadband, strongly coupled, and highly nonlinear, thereby motivating the development of new localization frameworks capable of effectively exploiting wideband measurement information.

Beyond the challenges posed by multi-band coupling and nonstationary dynamics, a more fundamental difficulty lies in the spatial characteristics of medium- and high-frequency oscillations [30]. Unlike low-frequency oscillations, which exhibit relatively coherent spatial patterns across the network, high-frequency oscillatory components tend to be highly localized due to impedance-induced attenuation [31,32]. As a result, source-related signatures are primarily manifested as local contrastive patterns rather than globally consistent responses.

However, most existing data-driven and graph-based localization methods implicitly assume smooth spatial dependencies and rely on neighbor aggregation mechanisms that emphasize global consistency. Such designs inherently introduce a low-pass filtering effect on graph signals, potentially suppressing critical high-frequency discriminative features and blurring local distinctions around oscillation sources.

Consequently, the key challenge in wideband oscillation localization is not only to model complex spatiotemporal dynamics, but also to preserve and enhance localized high-frequency contrast patterns that are essential for accurate source identification.

To address the aforementioned limitations, this paper proposes a novel spatiotemporal graph learning framework for wideband oscillation source localization.

Specifically, a dual-branch representation mechanism that integrates local contrast and global smoothness is developed to explicitly disentangle localized high-frequency perturbations from globally smooth structural responses.

In addition, a hierarchical channel interaction module is introduced to capture coupling relationships among multi-channel measurements both within each node and across the network, thereby enabling a more expressive representation of oscillatory dynamics.

By jointly modeling local contrast patterns and multi-channel interactions, the proposed framework effectively enhances the discriminability of source-related features in complex wideband oscillation scenarios.

It should be emphasized that the novelty of LCGS-Net does not lie in simply applying graph neural networks to oscillation source localization. Existing spatiotemporal graph learning methods usually rely on topology-based neighbor aggregation or graph attention to learn smooth spatial dependencies among buses, which is effective for low-frequency oscillations with coherent propagation patterns. However, such aggregation-oriented designs may suppress localized high-frequency perturbations and blur the discriminative differences between the true source bus and its electrically adjacent buses. In contrast, LCGS-Net is specifically designed from the spatial characteristics of wideband oscillations. It introduces a local-contrast/global-smooth dual-branch representation, where the global-smooth branch preserves network-level propagation context and the local-contrast branch explicitly enhances neighborhood discrepancies caused by localized medium- and high-frequency oscillatory components. Furthermore, the proposed framework integrates hierarchical multi-channel interaction and adaptive node-wise fusion, allowing the model to exploit heterogeneous voltage/current channel sensitivities and dynamically balance local contrastive features with global structural information. Therefore, compared with existing spatiotemporal graph learning methods, LCGS-Net provides a task-oriented and physically motivated representation framework for wideband oscillation source localization.

- A local-contrast-aware perspective for wideband oscillation source localization is proposed. Different from existing localization methods that mainly exploit globally coherent propagation patterns or smooth graph dependencies, this work highlights that medium- and high-frequency oscillations are often spatially localized and should be characterized by neighborhood contrast rather than only by global consistency.
- A local-contrast/global-smooth dual-branch graph representation module is developed. The global-smooth branch captures topology-aware propagation trends, while the local-contrast branch explicitly preserves high-frequency graph components and source-related neighborhood discrepancies. This design distinguishes LCGS-Net from conventional graph aggregation or graph attention models that mainly perform smoothing-based spatial feature propagation.
- A hierarchical multi-channel interaction and adaptive fusion mechanism is introduced to enhance source-related feature learning. The channel interaction module models the complementary sensitivities of different voltage/current measurement channels, while the adaptive fusion gate dynamically controls the contribution of local contrastive and global smooth representations for each node.
- Extensive simulations on power system test cases demonstrate that the proposed method significantly improves localization accuracy, particularly for medium- and high-frequency oscillations, under complex wideband and converter-dominated scenarios.

## 2 Methodology

### 2.1 Problem formulation

Consider a power network consisting of *N* buses, among which a subset $𝒮=\{s_{1},s_{2},...,s_{K}\}$ represents candidate oscillation sources corresponding to *K* grid-forming converter units (VSGs). Given multi-channel transient measurements collected from all buses, the objective of this work is to identify the oscillation source from the candidate set 𝒮 under wideband oscillatory conditions.

Let $𝐗\in {\mathbb{R}}^{N\times C\times T}$ denote the input measurement tensor, where *N* is the number of buses, *C* is the number of measurement channels per bus (voltage and current components), and *T* is the temporal length. For the *i*-th bus, its multi-channel time-series measurements are denoted as ${𝐗}_{i}\in {\mathbb{R}}^{C\times T}$.

The oscillation source localization task is formulated as a node-level classification problem over the candidate source set. Specifically, the goal is to learn a mapping function


$$\begin{array}{c} f:𝐗\to 𝐩, \end{array}$$ (1)

where $𝐩\in {\mathbb{R}}^{K}$ represents the predicted probability distribution over the *K* candidate source nodes.

For each sample, the ground-truth label is defined as a one-hot vector $𝐲\in {\mathbb{R}}^{K}$, where $y_{k}=1$ indicates that the *k*-th candidate node is the true oscillation source.

To facilitate graph-based modeling, the power network is represented as a graph 𝒢=(𝒱,ℰ), where 𝒱 is the set of buses and ℰ denotes the set of electrical connections. The corresponding adjacency matrix is denoted as $𝐀\in {\mathbb{R}}^{N\times N}$.

In wideband oscillation scenarios, the observed measurements exhibit both globally smooth propagation patterns and localized high-frequency perturbations. To capture these characteristics, the node representations are designed to encode both multi-channel temporal dynamics and spatial interactions across the graph. In particular, the key challenge lies in preserving localized contrastive features associated with oscillation sources while maintaining the global structural context of the network.

Based on the above formulation, the objective of this work is to learn a spatiotemporal representation model that can effectively extract discriminative features from 𝐗 and accurately identify the oscillation source node within the candidate set 𝒮.

### 2.2 Overall Architecture of the LCGS-Net (Local-Contrast Global-Smooth Network)

**Fig 1:**
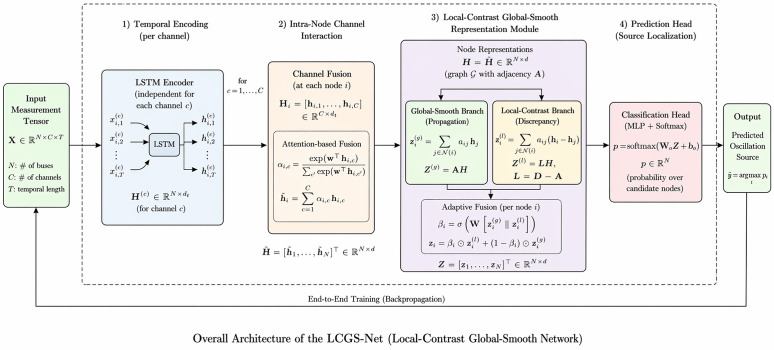
Architecture of the Local-Contrast Global-Smooth Network (LCGS-Net).

The overall architecture of the LCGS-Net is designed to effectively capture the spatiotemporal characteristics of wideband oscillations for source localization. As illustrated in Fig. 1, the model follows a hierarchical pipeline that integrates temporal dynamics modeling, intra-node multi-channel interaction, and graph-based spatial representation learning.

Given the input measurement tensor $𝐗\in {\mathbb{R}}^{N\times C\times T}$, where *N* denotes the number of buses, *C* is the number of measurement channels, and *T* is the temporal length, the framework first extracts temporal features for each channel independently. Specifically, for each node, the multi-channel time-series signals are processed by a temporal encoder based on long short-term memory (LSTM) networks, which are capable of capturing sequential dependencies and transient dynamics under nonstationary conditions. The outputs of the LSTM encoder summarize the temporal evolution of each channel and provide compact representations for subsequent processing.

After temporal encoding, an intra-node channel interaction module is introduced to model the coupling relationships among different measurement channels within each node. Instead of treating channels independently or simply concatenating them, this module enables information exchange across channels, allowing the model to capture complementary oscillatory signatures reflected in different electrical quantities.

The resulting node-level representations are then fed into a graph-based learning module constructed according to the physical topology of the power network. In this stage, a dual-branch representation mechanism is employed to explicitly model two distinct types of spatial patterns in wideband oscillations. The global-smooth branch captures the overall propagation trend of oscillatory responses across the network, reflecting the structural dependencies among buses. In contrast, the local-contrast branch emphasizes the discrepancies between neighboring nodes, which are particularly important for identifying localized high-frequency perturbations around oscillation sources.

To balance these two types of information, an adaptive fusion mechanism is adopted to combine the outputs of the two branches, generating refined node representations that simultaneously preserve global context and local discriminative features.

Finally, the learned representations are used to predict the oscillation source among the candidate nodes (VSG units) through a classification head. The overall framework thus enables effective extraction of source-related features by jointly modeling temporal dynamics, multi-channel interactions, and spatial contrast patterns in wideband oscillation scenarios.

### 2.3 Local-contrast and global-smooth representation module

In wideband oscillation scenarios, the spatial responses of the power network exhibit two fundamentally different characteristics. On the one hand, oscillatory disturbances propagate through the network following the physical topology, resulting in relatively smooth and correlated patterns across buses. On the other hand, due to impedance-induced attenuation and the localized nature of high-frequency components, the oscillation source and its neighboring nodes often exhibit pronounced discrepancies compared with the rest of the network. These observations indicate that effective source localization requires jointly modeling both globally smooth propagation patterns and locally contrastive features.

To this end, a dual-branch representation module is proposed to explicitly capture these two complementary aspects. Given the node-level representations $𝐇=[{𝐡}_{1},...,{𝐡}_{N}]\in {\mathbb{R}}^{N\times d}$ obtained from the previous stage, spatial interactions are modeled on the graph 𝒢 using two parallel branches.

The adjacency matrix is constructed from the physical network topology. Specifically, an initial binary adjacency matrix ${𝐀}_{0}$ is defined by transmission-line connections, where *A*_0,*ij*_ = 1 if bus *i* and bus *j* are directly connected, and *A*_0,*ij*_ = 0 otherwise. To preserve each bus’s own representation during graph propagation, self-loops are included by


$$\begin{array}{c} \bar{𝐀}={𝐀}_{0}+𝐈. \end{array}$$ (2)

Then, symmetric normalization is adopted:


$$\begin{array}{c} 𝐀={\bar{𝐃}}^{-\frac{1}{2}}\bar{𝐀}{\bar{𝐃}}^{-\frac{1}{2}}, \end{array}$$ (3)

where ${\bar{𝐃}}_{ii}=\sum_{j}{\bar{A}}_{ij}$ is the degree matrix of $\bar{𝐀}$. Accordingly, the normalized Laplacian used in the local-contrast branch is defined as


$$\begin{array}{c} 𝐋=𝐈-𝐀. \end{array}$$ (4)

Unless otherwise specified, 𝐀 in the following graph operations denotes the symmetrically normalized adjacency matrix with self-loops.

First, the global-smooth branch captures the overall propagation trend of oscillatory responses across the network. For each node, its representation is updated by aggregating information from neighboring nodes:


$$\begin{array}{c} {𝐳}_{i}^{\left(g\right)}=\sum_{j\in 𝒩(i)}a_{ij}{𝐡}_{j}, \end{array}$$ (5)

where $a_{ij}$ denotes the normalized adjacency weight. In matrix form, this process can be written as


$$\begin{array}{c} {𝐙}^{\left(g\right)}=𝐀𝐇, \end{array}$$ (6)

which emphasizes smooth spatial dependencies and shared oscillatory components.

In contrast, the local-contrast branch is designed to highlight discrepancies between nodes and their neighbors. Instead of directly aggregating neighbor features, this branch models the relative differences:


$$\begin{array}{c} {𝐳}_{i}^{\left(l\right)}=\sum_{j\in 𝒩(i)}a_{ij}({𝐡}_{i}-{𝐡}_{j}). \end{array}$$ (7)

This operation can be further interpreted as a graph Laplacian filtering process:


$$\begin{array}{c} {𝐙}^{\left(l\right)}=𝐋𝐇,\;𝐋=𝐃-𝐀, \end{array}$$ (8)

where 𝐋 is the graph Laplacian matrix and 𝐃 is the degree matrix. This formulation explicitly enhances high-frequency components in graph signals, thereby preserving localized perturbations associated with oscillation sources.

It should be noted that the Laplacian operation in the local-contrast branch is not claimed as a new graph filter by itself. Graph Laplacian filtering and spectral high-pass operations have been widely studied in graph signal processing, including graph wavelet networks, Chebyshev-polynomial-based spectral graph convolution, Bernstein-polynomial-based graph filters, signed-graph neural networks, and heterophilic graph neural networks [33–37]. The contribution of LCGS-Net lies in introducing this local-contrast operation into the specific task of wideband oscillation source localization and integrating it with a global-smooth branch, hierarchical channel interaction, and adaptive fusion. Different from graph wavelet or spectral filtering methods that mainly aim to design general-purpose graph filters, the proposed local-contrast branch is physically motivated by the localized attenuation characteristics of high-frequency oscillations in converter-dominated power systems. Different from signed-graph and heterophilic GNN methods that address label or feature heterophily on general graphs, LCGS-Net uses the Laplacian-based contrast as a source-discriminative pathway to highlight neighborhood discrepancies around oscillation sources, while the global-smooth branch simultaneously preserves the coherent propagation context of the power network. Therefore, the novelty of the proposed framework lies in its task-oriented dual-branch integration for wideband oscillation localization rather than in proposing a new standalone graph high-pass filter.

To adaptively balance the contributions of the two branches, a gating-based fusion mechanism is employed. For each node, the fusion coefficient is computed as


$$\begin{array}{c} \beta_{i}=\sigma \left(𝐖\left[{𝐳}_{i}^{\left(g\right)}\;\|\;{𝐳}_{i}^{\left(l\right)}\right]\right), \end{array}$$ (9)

and the final representation is given by


$$\begin{array}{c} {𝐳}_{i}=\beta_{i}⊙{𝐳}_{i}^{\left(l\right)}+(1-\beta_{i})⊙{𝐳}_{i}^{\left(g\right)}. \end{array}$$ (10)

In this implementation, $\beta_{i}$ is a learnable node-wise fusion gate generated from the concatenation of the global-smooth representation ${𝐳}_{i}^{\left(g\right)}$ and the local-contrast representation ${𝐳}_{i}^{\left(l\right)}$. The trainable projection matrix 𝐖 controls how the two branch features are mapped to the fusion coefficient, while the sigmoid function constrains $\beta_{i}$ to the range of (0,1). A larger value of $\beta_{i}$ assigns more weight to the local-contrast branch, indicating that localized high-frequency perturbations are more informative for the current node. Conversely, a smaller value of $\beta_{i}$ increases the contribution of the global-smooth branch, indicating that the coherent propagation pattern is more useful. Therefore, the balance between local and global information is not manually fixed, but is adaptively learned from data through the trainable gating parameters.

Through this design, the proposed module jointly captures global structural context and localized high-frequency perturbations. Compared with conventional graph aggregation methods, which tend to smooth node representations, the proposed approach effectively alleviates the over-smoothing effect and enhances the discriminability of source-related features.

In practice, the disentanglement is achieved by applying two different graph operators to the same node representation 𝐇. The global-smooth branch uses the normalized adjacency-based aggregation ${𝐙}^{\left(g\right)}=𝐀𝐇$, which averages neighboring node features and therefore extracts the low-frequency or spatially smooth components of the graph signal. In contrast, the local-contrast branch uses the Laplacian-based operation ${𝐙}^{\left(l\right)}=𝐋𝐇$, which computes the difference between each node and its neighbors and therefore emphasizes high-frequency graph components and localized perturbations. Thus, the two branches separate the input representation from complementary spatial perspectives: the global-smooth branch retains the coherent propagation trend across the network, whereas the local-contrast branch highlights source-related neighborhood discrepancies. The adaptive fusion gate then combines these two representations according to their relative importance for each node, enabling the model to preserve both global context and local high-frequency discriminative information.

### 2.4 Hierarchical channel interaction module

In wideband oscillation scenarios, the system dynamics are reflected through multiple measurement channels at each node, such as voltage and current components. These channels exhibit different sensitivities to oscillatory behavior across frequency bands and often provide complementary information for identifying oscillation sources. Therefore, treating each channel independently or simply concatenating them may lead to suboptimal feature representation.

To address this issue, a hierarchical channel interaction module is developed to explicitly model the coupling relationships among multi-channel measurements. The proposed design consists of two levels: intra-node channel interaction and inter-node channel-aware propagation.

#### 2.4.1 Intra-node channel interaction.

For each node *i*, let ${𝐇}_{i}=[{𝐡}_{i,1},...,{𝐡}_{i,C}]\in {\mathbb{R}}^{C\times d}$ denote the channel-wise representations. To capture channel dependencies, a learnable fusion function is applied:


$$\begin{array}{c} {\tilde{𝐡}}_{i}=\phi ({𝐇}_{i}), \end{array}$$ (11)

where ϕ(·) represents a parameterized mapping (e.g., linear projection or attention-based fusion). In practice, this operation can be implemented as


$$\begin{array}{c} {\tilde{𝐡}}_{i}=\sum_{c=1}^{C}\alpha_{i,c}{𝐡}_{i,c},\;\alpha_{i,c}=\frac{\exp({𝐰}^{⊤}{𝐡}_{i,c})}{\sum_{c^{\prime }}\exp({𝐰}^{⊤}{𝐡}_{i,c^{\prime }})}, \end{array}$$ (12)

where $\alpha_{i,c}$ denotes the importance weight of the *c*-th channel. This formulation enables adaptive selection of informative channels under different oscillation conditions.

#### 2.4.2 Inter-node channel-aware propagation.

Beyond intra-node coupling, oscillatory information propagates across the network in a channel-dependent manner. To incorporate this effect, the node representations obtained after channel interaction are propagated over the graph:


$$\begin{array}{c} \tilde{𝐙}=𝐀\tilde{𝐇}, \end{array}$$ (13)

where $\tilde{𝐇}=[{\tilde{𝐡}}_{1},...,{\tilde{𝐡}}_{N}{]}^{⊤}$. This operation allows the model to capture how channel-combined features interact across neighboring nodes.

#### 2.4.3 Integration with dual-branch representation.

The resulting node representations $\tilde{𝐇}$ are then fed into the dual-branch module described in Section 2.3. By combining channel-aware feature learning with local-contrast and global-smooth modeling, the proposed framework is able to effectively capture both multi-channel coupling and spatial discriminative patterns.

In summary, the hierarchical channel interaction module enhances feature representation by incorporating both intra-node channel dependencies and inter-node propagation effects, which is crucial for modeling wideband oscillation dynamics.

More specifically, the hierarchical channel interaction module follows a two-stage architecture. In the first stage, channel-wise temporal embeddings at each bus are processed by an intra-node attention mechanism to obtain a channel-adaptive node representation. This allows the model to assign different importance weights to voltage and current channels according to their oscillation sensitivity. In the second stage, the channel-fused node representations are propagated through the physical network topology, so that the channel-aware oscillation features can interact across electrically connected buses. Compared with standard GNN layers that usually aggregate a single node feature vector from neighboring nodes, the proposed module performs channel interaction before graph propagation. Therefore, it can preserve the complementary information carried by different measurement channels and avoid prematurely compressing heterogeneous voltage/current responses into an indistinguishable node feature. This design improves feature learning by enabling the subsequent graph module to operate on oscillation-sensitive and channel-adaptive node representations.

### 2.5 Loss function

The oscillation source localization task is formulated as a classification problem over the candidate source set. Given the predicted probability vector $𝐩\in {\mathbb{R}}^{K}$ and the ground-truth one-hot label $𝐲\in {\mathbb{R}}^{K}$, the model is trained using the standard cross-entropy loss.

Specifically, the loss for a single sample is defined as


$$\begin{array}{c} ℒ=-\sum_{k=1}^{K}y_{k}\log p_{k}, \end{array}$$ (14)

where $p_{k}$ denotes the predicted probability that the *k*-th candidate node is the oscillation source.

For a dataset containing *M* samples, the overall training objective is given by


$$\begin{array}{c} {ℒ}_{\text{total}}=\frac{1}{M}\sum_{m=1}^{M}{ℒ}^{\left(m\right)}. \end{array}$$ (15)

This formulation does not introduce additional hyperparameters and provides a stable and effective objective for training the proposed model.

## 3 Experiments and results

### 3.1 Dataset construction

**Fig 2:**
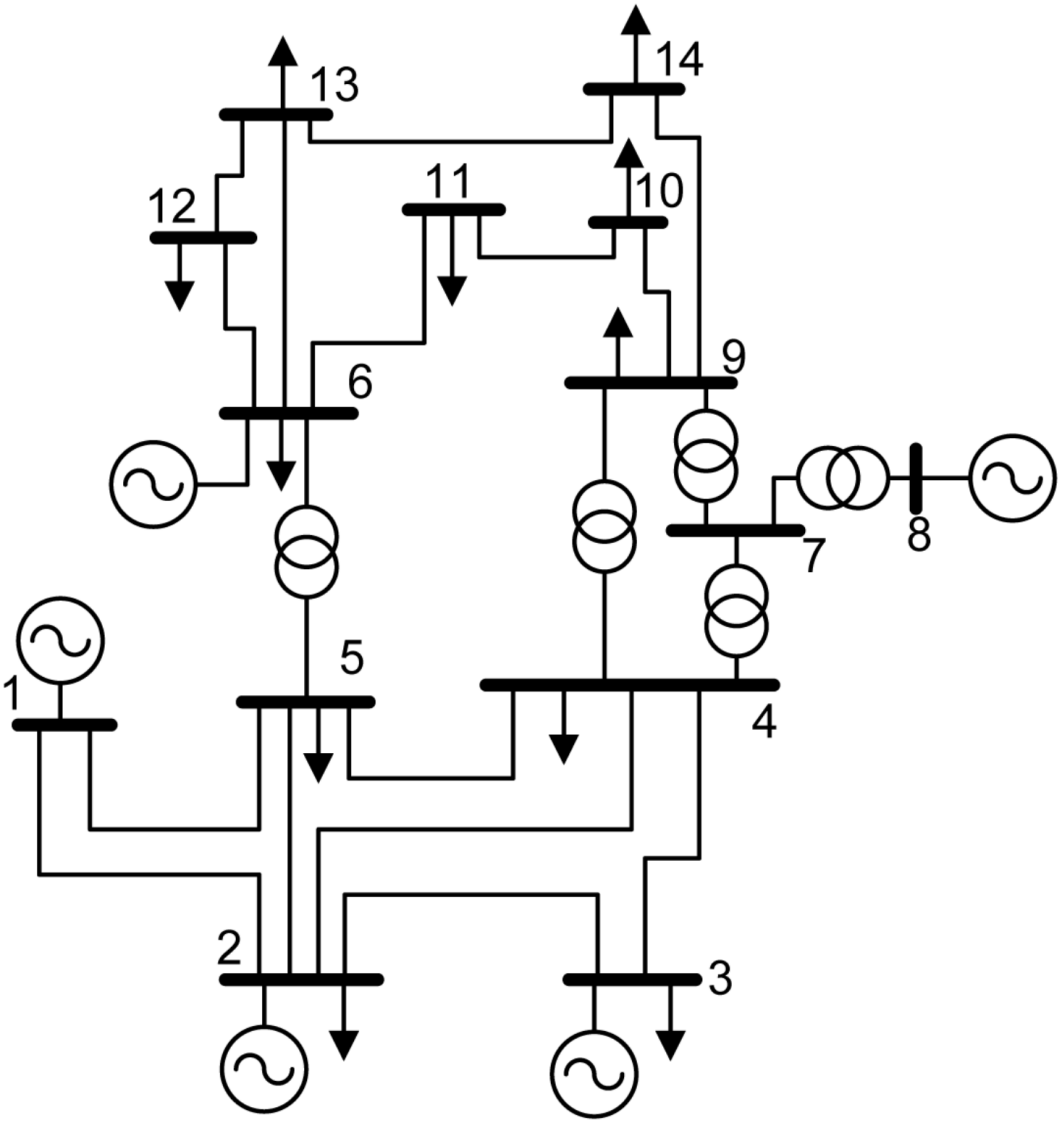
IEEE 14-bus test system with integrated VSG units and measurement locations.

To evaluate the effectiveness of the proposed method, a dataset is constructed based on time-domain simulations of the IEEE 14-bus test system implemented in MATLAB/Simulink. The system topology is illustrated in Fig 2, where multiple converter-interfaced units (VSGs) are integrated into selected buses.

The IEEE 14-bus system is selected as the primary benchmark because it provides a simple and controlled network topology for verifying the basic effectiveness of the proposed localization framework under converter-interfaced oscillation scenarios. In this study, the main objective is to validate the proposed local-contrast and global-smooth representation mechanism in a small-scale benchmark system rather than to claim full representativeness of large practical converter-dominated grids. To further examine the scalability of the proposed method, additional preliminary experiments on the IEEE 39-bus system are also conducted.

In the simulation setup, five buses equipped with VSG units are considered as candidate oscillation sources. For each sample, a wideband oscillation disturbance is injected at one of these candidate nodes. The injected oscillation consists of multiple frequency components, including low-frequency, medium-frequency, and high-frequency components, in order to emulate realistic wideband oscillatory behaviors observed in converter-dominated power systems.

For each sample, a wideband oscillation disturbance is injected at one of these candidate nodes. In this study, the injected oscillation signal is mathematically represented as a damped sinusoidal disturbance:


$$\begin{array}{c} u_{osc}(t)=Ae^{-\xi t}\sin(2\pi ft+\phi ), \end{array}$$ (16)

where *A*, ξ, *f*, and ϕ denote the oscillation amplitude, damping coefficient, oscillation frequency, and initial phase, respectively. According to the frequency setting used in the simulations, the oscillation scenarios are divided into two groups using 50 Hz as the boundary. Specifically, oscillations with *f* < 50 Hz are defined as low-frequency oscillation scenarios, while oscillations with *f* > 50 Hz are defined as high-frequency oscillation scenarios. This division is used to evaluate the localization performance of different methods under globally more coherent low-frequency oscillations and more localized high-frequency oscillations.

For each simulation case, multi-channel measurements are collected from all *N* = 14 buses. The recorded signals include voltage and current measurements at each bus, forming a multi-channel time-series dataset. These measurements capture the spatiotemporal propagation of oscillatory disturbances across the network.

To simulate practical scenarios with limited measurement availability, only a subset of buses is assumed to be observable during training and testing. Specifically, for each sample, approximately half of the buses are randomly selected as observed nodes, while measurements from the remaining buses are masked out. This setting reflects realistic partial observability conditions in wide-area monitoring systems, where measurement devices may only be installed at limited locations.

Specifically, half of the available channels are randomly selected for each sample, while the remaining channels are discarded. This setting reflects incomplete sensing conditions and evaluates the robustness of the proposed method under partial observability.

A total of 10000 samples are generated under different disturbance configurations and source locations. The dataset is randomly divided into training, validation, and test sets with a ratio of 8:1:1. All samples are generated through independent simulations to ensure diversity in oscillation patterns and operating conditions.

### 3.2 Implementation details

To improve the reproducibility of the proposed method, the main training hyperparameters and model configurations used in the experiments are summarized in Table 1. All compared models are trained using the same training, validation, and test splits. The validation set is used for hyperparameter selection and early stopping, while the test set is only used for final performance evaluation.

**Table 1:** Implementation details and training hyperparameters of LCGS-Net.

Item	Setting
Optimizer	Adam
Initial learning rate	$1.0\times 10^{-3}$
Weight decay	$1.0\times 10^{-5}$
Batch size	64
Maximum number of epochs	100
Early stopping patience	15 epochs
Loss function	Cross-entropy loss
LSTM hidden dimension	128
Number of LSTM layers	4
Node hidden dimension	128
Dropout rate	0.2
Activation function	ReLU
Number of candidate sources on IEEE 14-bus	5
Number of candidate sources on IEEE 39-bus	5/7/10
Train/validation/test split	8:1:1

For a fair comparison, the baseline models are trained under the same optimizer, batch size, maximum epoch number, early-stopping criterion, and data split. Model-specific hidden dimensions and layer numbers are tuned on the validation set to obtain their best performance. No test-set information is used during hyperparameter tuning.

### 3.3 Performance metrics

The oscillation source localization task is formulated as a multi-class classification problem over the candidate source nodes. Accordingly, standard classification metrics are adopted to evaluate the performance of the proposed method.

#### 3.3.1 Accuracy.

Accuracy is used as the primary evaluation metric, which measures the proportion of correctly identified oscillation sources:


$$\begin{array}{c} \text{Accuracy}=\frac{1}{M}\sum_{m=1}^{M}𝕀\left({\hat{k}}^{\left(m\right)}=k^{\left(m\right)}\right), \end{array}$$ (17)

where *M* is the number of test samples, ${\hat{k}}^{\left(m\right)}$ and *k*^(*m*)^ denote the predicted and true source indices for the *m*-th sample, respectively, and 𝕀(·) is the indicator function.

#### 3.3.2 Macro-F1 score.

To further evaluate the classification performance across different source nodes, the Macro-F1 score is adopted. For each class, the precision and recall are defined as


$$\begin{array}{c} {\text{Precision}}_{k}=\frac{TP_{k}}{TP_{k}+FP_{k}},\;{\text{Recall}}_{k}=\frac{TP_{k}}{TP_{k}+FN_{k}}, \end{array}$$ (18)

where $TP_{k}$, $FP_{k}$, and $FN_{k}$ denote the number of true positives, false positives, and false negatives for class *k*, respectively.

The F1-score for class *k* is computed as


$$\begin{array}{c} {\text{F1}}_{k}=\frac{2\cdot {\text{Precision}}_{k}\cdot {\text{Recall}}_{k}}{{\text{Precision}}_{k}+{\text{Recall}}_{k}}. \end{array}$$ (19)

The Macro-F1 score is then obtained by averaging over all *K* classes:


$$\begin{array}{c} \text{Macro-F1}=\frac{1}{K}\sum_{k=1}^{K}{\text{F1}}_{k}. \end{array}$$ (20)

This metric treats all classes equally and is particularly useful for evaluating performance under potential class imbalance conditions.

Overall, Accuracy and Macro-F1 provide a comprehensive and standard evaluation of the proposed method from both overall correctness and class-wise performance perspectives.

It should be noted that, in this closed-set source localization task, the Accuracy metric is equivalent to the Top-1 hit rate, because a prediction is counted as correct only when the predicted candidate source node is exactly the same as the true source node. To further quantify the severity of incorrect localization, a graph-distance-based localization error is also considered:


$$\begin{array}{c} E_{dist}=\frac{1}{M}\sum_{m=1}^{M}d_{𝒢}\left({\hat{s}}^{\left(m\right)},s^{\left(m\right)}\right), \end{array}$$ (21)

where ${\hat{s}}^{\left(m\right)}$ and *s*^(*m*)^ denote the predicted and true source nodes of the *m*-th test sample, respectively, and $d_{𝒢}(\cdot ,\cdot )$ represents the shortest-path distance between two buses on the network graph. A smaller $E_{dist}$ indicates that the incorrectly localized source is electrically closer to the true source. Therefore, the proposed method is quantitatively evaluated using Top-1 hit rate/Accuracy, Macro-F1, and graph-distance-based localization error.

### 3.4 Comparison with existing methods

**Fig 3:**
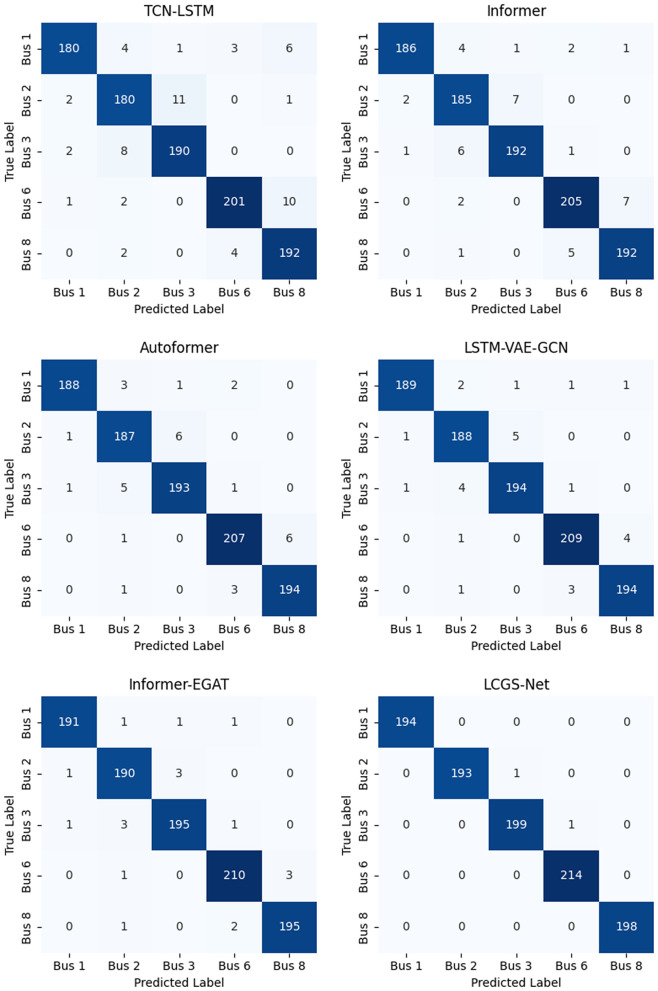
Confusion matrices of different methods under sub-synchronous low-frequency oscillation scenarios.

To evaluate the effectiveness of the proposed method, comparative experiments are conducted under sub-synchronous low-frequency oscillation scenarios. Several representative baselines are considered, including conventional sequence-based models (TCN-LSTM [38], Informer [39], and Autoformer [40]) as well as graph-enhanced oscillation localization methods (LSTM-VAE-GCN [23] and Informer-EGAT [24]). The localization accuracy of different methods is summarized in Table 2, and the corresponding confusion matrices are shown in Fig. 3.

**Table 2:** Comparison of localization accuracy under sub-synchronous low-frequency oscillation scenarios.

Method	Accuracy (%)
TCN-LSTM	94.3
Informer	96.0
Autoformer	96.9
LSTM-VAE-GCN	97.4
Informer-EGAT	98.1
**LCGS-Net**	**99.8**

These baseline models are selected to cover both temporal sequence learning methods and graph-enhanced spatiotemporal localization methods. For a fair comparison, all models are trained and evaluated using the same training, validation, and test splits, the same input measurement channels, the same source labels, and the same performance metrics. The key hyperparameters of each baseline, including hidden dimension, number of layers, learning rate, batch size, dropout rate, and training epochs, are tuned on the validation set. No test-set information is used during hyperparameter selection. All models are trained under the same optimizer and early-stopping strategy to ensure that the comparison reflects differences in model architecture rather than differences in experimental settings.

As shown in Table 2, the proposed LCGS-Net achieves the highest localization accuracy of 99.8%, outperforming all compared baseline methods. Among the conventional sequence-based models, TCN-LSTM achieves 94.3%, while Informer and Autoformer obtain 96.0% and 96.9%, respectively. After incorporating topology-aware spatial modeling, LSTM-VAE-GCN further improves the localization accuracy to 97.4%, and Informer-EGAT achieves 98.1% through joint spatiotemporal attention modeling. Nevertheless, the proposed LCGS-Net still achieves the best overall performance.

The confusion matrices in Fig 3 provide further insights into the behavior of different methods. For TCN-LSTM, noticeable misclassification can be observed between neighboring buses, especially between Bus 2 and Bus 3, as well as Bus 6 and Bus 8. Although Informer and Autoformer improve the overall classification performance by capturing longer temporal dependencies, residual confusion among electrically adjacent buses still exists.

Compared with purely temporal sequence models, graph-enhanced methods such as LSTM-VAE-GCN and Informer-EGAT exhibit better localization capability. By incorporating network topology information and spatial interactions, these methods are able to reduce the confusion among neighboring nodes and achieve more structured source representations. However, a small number of misclassifications can still be observed in the confusion matrices, indicating that conventional graph aggregation mechanisms may still smooth out subtle node-level differences during feature propagation.

In contrast, the proposed LCGS-Net produces almost perfect classification results, with only negligible misclassifications across all source nodes. This improvement can be attributed to the proposed local-contrast and global-smooth dual-branch representation mechanism. The global-smooth branch captures the overall propagation trend and structural dependency of oscillatory responses, while the local-contrast branch explicitly preserves neighborhood discrepancies and enhances source-related discriminative features. Such a design effectively alleviates the over-smoothing effect commonly encountered in conventional graph-based representations.

Furthermore, the hierarchical channel interaction module enables adaptive modeling of heterogeneous channel sensitivities across different observed buses, thereby improving the representation capability of multi-channel oscillation measurements.

Overall, the experimental results demonstrate that the proposed framework is able to more effectively capture both temporal dynamics and spatial discriminative characteristics of oscillatory propagation, leading to superior localization performance compared with existing sequence-based and graph-based methods.

### 3.5 Performance under high-frequency oscillation scenarios

To further evaluate the effectiveness of the proposed framework under more challenging conditions, additional comparative experiments are conducted on a newly constructed dataset containing high-frequency oscillation scenarios. Different from the sub-synchronous low-frequency dataset, the high-frequency dataset is independently generated, and the numbers of samples corresponding to different source nodes are not strictly identical. The total number of test samples is approximately 1000.

**Fig 4:**
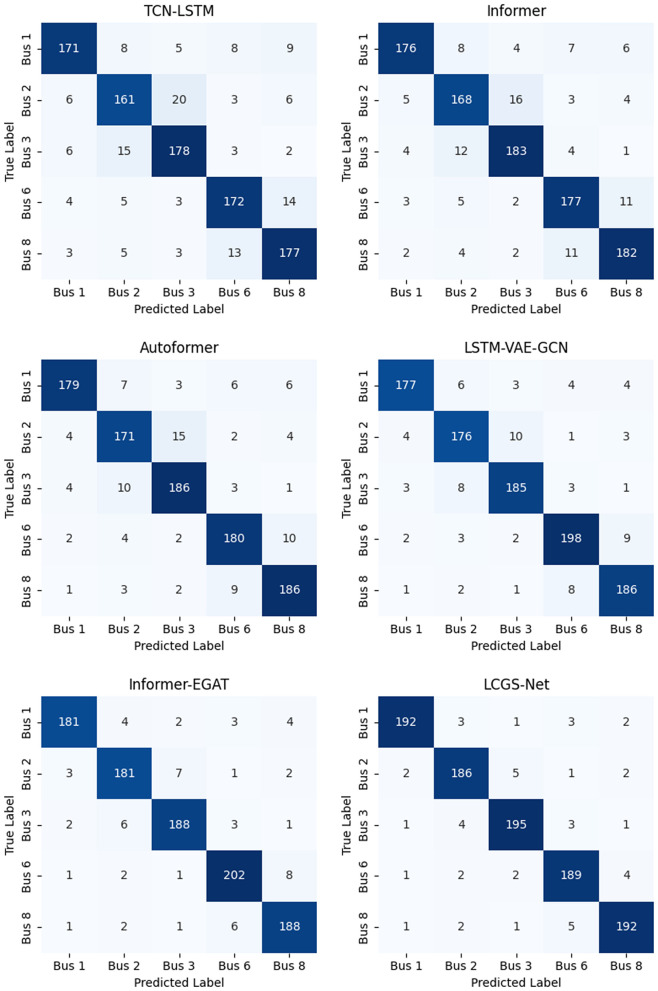
Confusion matrices of different methods under high-frequency oscillation scenarios.

Compared with low-frequency oscillations, high-frequency oscillations exhibit significantly stronger locality, faster spatial attenuation, and more complex propagation characteristics, making oscillation source localization substantially more challenging. The localization performance of different methods is summarized in Table 3, and the corresponding confusion matrices are shown in Fig 4. It can be observed that the performance of all methods decreases compared with the low-frequency scenario, confirming the increased difficulty of high-frequency oscillation localization.

**Table 3:** Performance comparison under high-frequency oscillation scenarios.

Method	Accuracy (%)	Macro-F1 (%)
TCN-LSTM	85.9	85.6
Informer	88.6	88.3
Autoformer	90.2	90.0
LSTM-VAE-GCN	92.2	92.0
Informer-EGAT	94.0	93.9
**LCGS-Net**	**95.4**	**95.3**

As shown in Table 3, the proposed LCGS-Net achieves the highest localization accuracy of 95.4% and the highest Macro-F1 score of 95.3%, outperforming all compared baseline methods under high-frequency oscillation scenarios. Among the conventional sequence-based models, TCN-LSTM achieves 85.9%, while Informer and Autoformer obtain 88.6% and 90.2%, respectively. After incorporating graph-aware spatial modeling, LSTM-VAE-GCN improves the accuracy to 92.2%, and Informer-EGAT further achieves 94.0% through spatiotemporal graph attention modeling. Nevertheless, the proposed LCGS-Net still achieves the best overall localization performance.

The confusion matrices in Fig 4 further reveal the challenges of high-frequency oscillation localization. Compared with the low-frequency scenario, the confusion among neighboring buses becomes significantly more severe for all baseline methods, especially between Bus 2 and Bus 3, as well as Bus 6 and Bus 8. This phenomenon indicates that high-frequency oscillatory responses exhibit much weaker global coherence and stronger localized perturbation characteristics.

For conventional sequence-based models such as TCN-LSTM, Informer, and Autoformer, the localization performance degrades substantially under high-frequency conditions. Although these methods are effective in capturing temporal dependencies, they mainly focus on global temporal similarities and lack explicit modeling of localized spatial discrepancies. Consequently, they are less capable of distinguishing source nodes with highly similar oscillatory propagation patterns.

Compared with purely temporal models, graph-enhanced methods such as LSTM-VAE-GCN and Informer-EGAT exhibit improved localization performance by incorporating topology-aware spatial interactions. However, residual confusion among electrically adjacent nodes can still be observed. This suggests that conventional graph aggregation and graph attention mechanisms still tend to emphasize smooth spatial propagation patterns, which may weaken localized high-frequency perturbations during feature propagation.

In contrast, the proposed LCGS-Net still maintains strong discrimination capability under high-frequency conditions. This improvement mainly benefits from the proposed local-contrast and global-smooth dual-branch representation mechanism. The local-contrast branch explicitly enhances neighborhood discrepancies and preserves localized perturbation patterns associated with high-frequency oscillations, while the global-smooth branch simultaneously maintains the structural context of oscillation propagation across the network. By jointly modeling local contrastive features and global structural dependencies, the proposed framework effectively alleviates the over-smoothing problem commonly encountered in conventional graph-based methods.

Furthermore, the hierarchical channel interaction module enables adaptive modeling of heterogeneous channel sensitivities across different observed buses, thereby improving the representation capability of multi-channel oscillation measurements under partial observability conditions.

Overall, the experimental results under high-frequency oscillation scenarios demonstrate that the proposed framework is particularly suitable for wideband oscillation source localization, especially when oscillatory dynamics exhibit strong locality, rapid attenuation, and weak global coherence.

### 3.6 Computational cost analysis

To evaluate the computational feasibility of the proposed LCGS-Net for online oscillation monitoring, a computational cost comparison is conducted on the IEEE 14-bus dataset. The parameter count, training time per epoch, and single-sample inference time are reported for LCGS-Net and the compared baseline models. All models are evaluated under the same hardware and software environment, and the inference time is measured as the average processing time for one test sample.

As shown in Table 4, LCGS-Net has 0.563 million trainable parameters, which is smaller than all compared baseline models. Although the proposed model introduces local-contrast/global-smooth dual-branch graph representation and hierarchical channel interaction, these modules are mainly based on lightweight graph operations and attention-based channel fusion. Therefore, the overall model size remains moderate.

**Table 4:** Computational cost comparison on the IEEE 14-bus dataset.

Method	Parameters (M)	Training time (s/epoch)	Inference time (ms/sample)
TCN-LSTM	0.742	5.18	0.71
Informer	1.286	7.42	1.36
Autoformer	1.374	7.86	1.49
LSTM-VAE-GCN	0.968	6.54	1.08
Informer-EGAT	1.684	9.31	1.84
**LCGS-Net**	**0.563**	**5.02**	**0.86**

In terms of training efficiency, LCGS-Net requires 5.02 s per epoch, which is comparable to TCN-LSTM and lower than the Transformer-based and graph-enhanced baselines. For online monitoring, the most critical indicator is the single-sample inference time. LCGS-Net achieves an average inference time of 0.86 ms/sample, which is faster than Informer, Autoformer, LSTM-VAE-GCN, and Informer-EGAT. This millisecond-level inference time indicates that the proposed framework has the potential to support real-time or near-real-time oscillation source localization in online monitoring scenarios.

It should be noted that the reported computational cost is measured on the benchmark simulation dataset. When deployed in practical large-scale grids, additional computational overhead may be introduced by PMU data preprocessing, communication delay handling, bad-data detection, and larger graph topology processing. Therefore, further deployment-oriented optimization and hardware-specific acceleration will be considered in future work.

### 3.7 Visualization and interpretability analysis

To further validate the effectiveness and interpretability of the proposed method, several visualization experiments are conducted. A representative case is selected where the oscillation source is located at Bus 6, and the learned representations and model responses are analyzed from multiple perspectives.

#### 3.7.1 Predicted source probability distribution.

**Fig 5:**
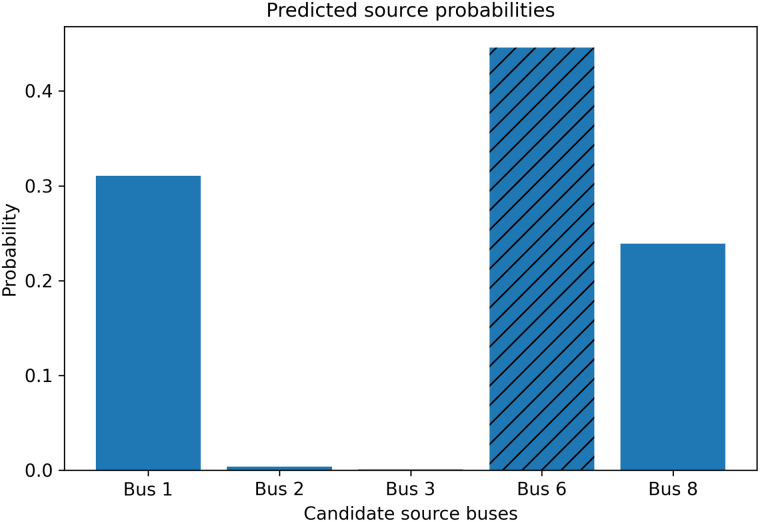
Predicted source probability distribution for a sample with source at Bus 6.

Fig 5 illustrates the predicted probability distribution over candidate source nodes. It can be observed that the proposed model assigns the highest probability to Bus 6, which is consistent with the ground truth. Meanwhile, relatively lower probabilities are assigned to other nodes, such as Bus 1 and Bus 8, which are topologically close to the source.

This result demonstrates that the model is able to effectively distinguish the true source from its neighboring nodes, rather than producing ambiguous or overly smooth predictions. Such discriminative capability is mainly attributed to the local-contrast mechanism, which explicitly enhances the differences between a node and its neighbors.

#### 3.7.2 Representation analysis of dual-branch features.

**Fig 6:**
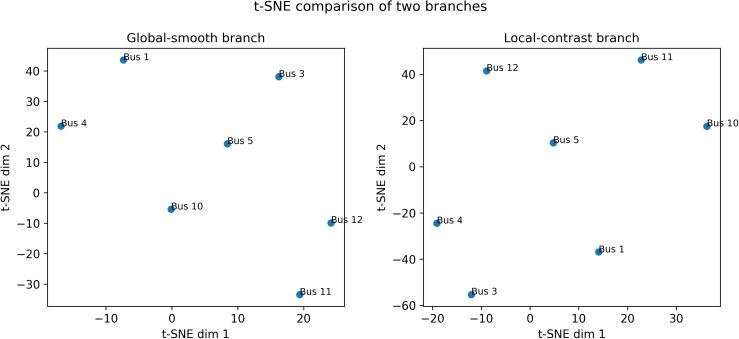
t-SNE visualization of node representations from the global-smooth and local-contrast branches.

To investigate how the proposed dual-branch representation contributes to source localization, t-SNE visualization is applied to the learned node embeddings from the global-smooth branch and the local-contrast branch, as shown in Fig 6.

From the global-smooth branch, the node representations exhibit relatively structured and topology-aware distributions. Nodes that are electrically correlated through the network tend to form smoother spatial arrangements in the embedding space. For example, Bus 5, Bus 10, and Bus 12 remain relatively close to the central representation region, indicating that the global-smooth branch mainly captures the large-scale propagation behavior and coherent spatial dependencies of oscillatory dynamics.

This phenomenon is consistent with the graph aggregation operation in Eq. (3), where neighboring node information is recursively propagated and averaged through the adjacency matrix. Such a mechanism naturally emphasizes low-frequency graph components and smooth structural correlations across the network.

In contrast, the local-contrast branch produces significantly more separated node representations. Compared with the global-smooth branch, several nodes become more distinguishable in the embedding space, especially those associated with localized oscillatory behaviors. For instance, Bus 11 and Bus 12 exhibit clear spatial separation from other nodes, while Bus 10 is pushed toward another distinct region. This indicates that the local-contrast branch effectively amplifies local discrepancies among neighboring buses.

Such behavior is physically consistent with the characteristics of medium- and high-frequency oscillations in converter-dominated systems. Due to impedance-induced attenuation, high-frequency oscillatory energy tends to remain spatially localized around the source region rather than propagating coherently across the entire network. Consequently, neighboring buses may exhibit significantly different transient signatures even when they are topologically close.

The observed representation separation can be further explained by the graph Laplacian operation in Eq. (5). Unlike conventional graph aggregation, the Laplacian-based contrastive filtering explicitly extracts high-frequency graph components by emphasizing the relative differences between a node and its neighbors. As a result, localized perturbation patterns that would otherwise be smoothed out by traditional graph convolution can be effectively preserved and enhanced.

Moreover, comparing the two branches reveals their complementary roles. The global-smooth branch mainly provides structural context and captures the overall oscillation propagation trend, while the local-contrast branch focuses on localized discriminative perturbations that are critical for source identification under wideband oscillation conditions. Their combination enables the proposed framework to simultaneously preserve network-level coherence and source-level discriminability.

Overall, the visualization results strongly support the motivation of the proposed dual-branch design and verify that localized high-frequency contrast patterns constitute an important representation characteristic for wideband oscillation source localization.

#### 3.7.3 Aggregated representation visualization across test samples.

**Fig 7:**
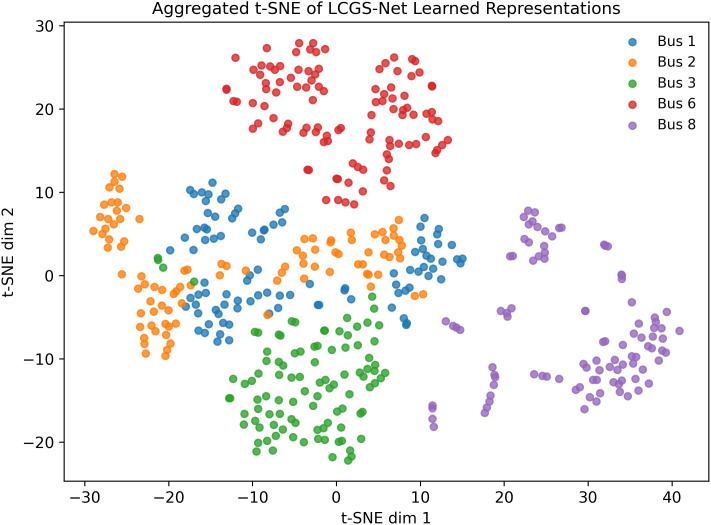
Aggregated t-SNE visualization of LCGS-Net learned representations across multiple test samples.

To provide a more representative visualization of the learned feature space, an aggregated t-SNE analysis is further conducted across multiple test samples. Different from the previous single-sample visualization, the learned representations from the test set are collected and projected into a two-dimensional space using t-SNE. Each point in Fig 7 represents one learned representation associated with a test sample, and different colors correspond to different candidate oscillation source buses.

As shown in Fig 7, the learned representations of different source classes form relatively distinguishable clusters in the embedding space. For example, the representations associated with Bus 6 and Bus 8 are clearly separated from most other classes, indicating that LCGS-Net learns discriminative source-related features rather than relying on a single accidental sample pattern. Although partial overlap can still be observed among some electrically adjacent buses, such as Bus 1, Bus 2, and Bus 3, the overall class-wise structure remains evident. This phenomenon is reasonable because neighboring buses may exhibit similar oscillation propagation responses, especially under partial observability and wideband disturbance conditions.

The aggregated visualization further supports the effectiveness of the proposed local-contrast/global-smooth representation mechanism. The local-contrast branch enhances neighborhood discrepancies associated with source-related perturbations, while the global-smooth branch preserves the structural propagation context across the network. As a result, the learned feature space exhibits both intra-class compactness and inter-class separability across multiple test samples. Compared with a single-case visualization, this aggregated t-SNE analysis provides stronger evidence that the proposed model consistently learns discriminative representations for oscillation source localization.

#### 3.7.4 Channel attention analysis.

**Fig 8:**
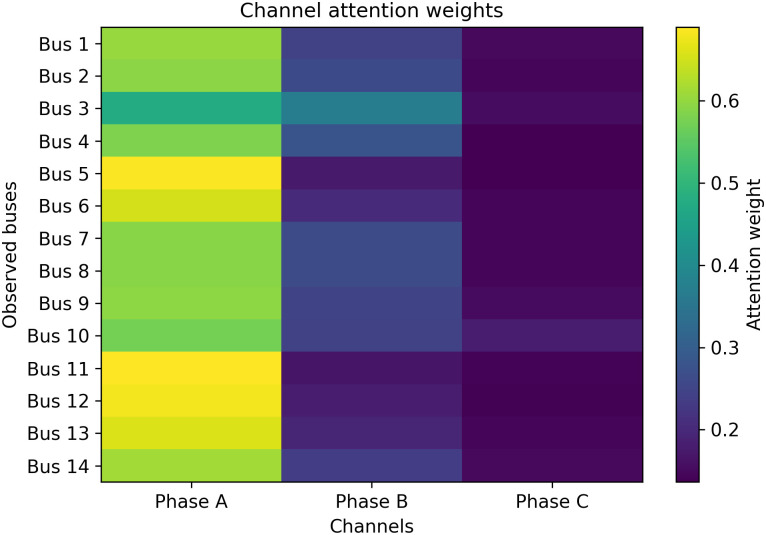
Average channel attention weights across observed buses on the test set.

Fig 8 presents the average channel attention weights across the test set for different observed buses under the wideband oscillation scenario. Different from the original single-example visualization, this figure summarizes the overall channel attention distribution learned by the model over all test samples, and therefore provides a more statistically representative interpretation of the channel interaction mechanism.

It can be observed that the attention distributions vary across both buses and channels, indicating that different measurement channels exhibit different sensitivities to oscillatory dynamics at different network locations. In particular, Phase A generally receives the largest attention weights for most observed buses, while Phase B shows moderate contributions and Phase C receives relatively smaller attention weights. Several buses, such as Bus 5, Bus 11, and Bus 12, exhibit especially strong responses on Phase A, whereas other buses show relatively more balanced channel responses.

This phenomenon suggests that oscillatory perturbations are not uniformly reflected across all measurement channels, but instead manifest differently depending on local electrical conditions and oscillation propagation characteristics. Such behavior is physically consistent with converter-dominated wideband oscillations, where impedance-induced attenuation, asymmetric converter interactions, and localized propagation effects may cause oscillation energy to become more observable in certain channels than in others. Consequently, the average channel sensitivity distributions exhibit clear spatial heterogeneity across the network.

It is important to note that the proposed channel interaction mechanism is not intended to simply perform weighted averaging across channels. Instead, the learned attention responses characterize the relative contribution and oscillation sensitivity of different measurement channels during feature extraction and representation learning. Since only a subset of buses is randomly selected as observable nodes during training and testing, the model is required to dynamically exploit informative channel patterns under partial observability conditions. The averaged attention visualization demonstrates that the proposed framework can still identify stable and oscillation-relevant channel-response patterns across the test set, even when the network measurements are incomplete.

Overall, the results verify that the proposed hierarchical channel interaction module effectively captures heterogeneous and location-dependent channel sensitivities, thereby enhancing the representation capability and robustness of wideband oscillation source localization.

#### 3.7.5 Discussion.

Overall, the visualization results provide strong evidence for the effectiveness of the proposed design. The probability distribution confirms accurate source identification, the t-SNE analysis demonstrates the complementary roles of the dual-branch representation, and the channel attention map reveals adaptive multi-channel feature utilization.

These findings validate that the proposed framework not only achieves high predictive performance but also captures meaningful physical and structural characteristics of wideband oscillations, thereby enhancing both interpretability and reliability.

### 3.8 Ablation study

To further investigate the contributions of different components in the proposed LCGS-Net, an ablation study is conducted under both low-frequency and high-frequency oscillation scenarios. The results are summarized in Table 5.

**Table 5:** Ablation study under low-frequency and high-frequency oscillation scenarios.

Method	Low-frequency		High-frequency	
	Accuracy (%)	Macro-F1 (%)	Accuracy (%)	Macro-F1 (%)
Full LCGS-Net	**99.8**	**99.8**	**95.4**	**95.3**
w/o Local-Contrast	97.2	97.0	90.8	90.5
w/o Global-Smooth	97.8	97.6	92.1	91.8
w/o Channel Interaction	98.4	98.2	93.0	92.7
w/o Adaptive Fusion	98.9	98.7	94.1	93.9

It can be observed that removing any component leads to a performance degradation, demonstrating that each module plays an important role in the overall framework. Among all variants, removing the local-contrast branch results in the most significant performance drop, especially under high-frequency oscillation scenarios, where the accuracy decreases from 95.4% to 90.8%. This clearly indicates that the local-contrast mechanism is crucial for capturing localized high-frequency perturbations and distinguishing the oscillation source from its neighboring nodes.

In contrast, removing the global-smooth branch also degrades the performance, but to a lesser extent. This suggests that global structural information still provides useful contextual guidance, although it is less critical than local contrast in high-frequency scenarios.

Furthermore, replacing the hierarchical channel interaction module with simple channel averaging leads to a noticeable performance reduction. This confirms that different measurement channels carry complementary information, and explicitly modeling their interactions enhances the representation capability.

Finally, replacing the adaptive fusion mechanism with fixed weighting also results in performance degradation, indicating that the relative importance of local and global features varies across different nodes and oscillation conditions. The learnable gating mechanism allows the model to dynamically balance these contributions, leading to improved robustness.

Comparing the results across frequency bands, it can be observed that the performance degradation is generally more pronounced under high-frequency scenarios. This highlights the increased difficulty of high-frequency oscillation localization and further demonstrates the effectiveness of the proposed modules in addressing this challenge.

Overall, the ablation results validate that the proposed local-contrast and global-smooth dual-branch design, together with hierarchical channel interaction and adaptive fusion, jointly contribute to the superior performance of LCGS-Net.

### 3.9 Robustness analysis under measurement disturbances

To further evaluate the robustness of the proposed LCGS-Net under practical measurement conditions, additional experiments are conducted by introducing different types of measurement disturbances into the IEEE 14-bus test dataset. In real wide-area monitoring systems, PMU or converter-side measurements may be affected by random noise, impulsive bad data, short-term missing values, or non-Gaussian measurement errors. Therefore, four representative disturbance conditions are considered in this study: Gaussian noise with a signal-to-noise ratio of 30 dB, impulsive noise with 1% corrupted sampling points, 5% missing data followed by interpolation, and Laplace noise with an equivalent 30 dB noise level. The model is evaluated under the same test protocol, and the results are summarized in Table 6.

**Table 6:** Robustness of LCGS-Net under different measurement disturbances on the IEEE 14-bus system.

Measurement condition	Accuracy (%)	Macro-F1 (%)
Clean measurements	96.8	96.6
Gaussian noise, 30 dB	94.7	94.4
Impulsive noise, 1% corrupted points	94.0	93.7
Missing data, 5% with interpolation	94.5	94.2
Laplace noise, equivalent 30 dB	94.2	93.9

As shown in Table 6, the proposed LCGS-Net maintains stable localization performance under different measurement disturbances. Compared with the clean-measurement condition, the accuracy decreases from 96.8% to 94.7% under Gaussian noise with 30 dB SNR and to 94.2% under Laplace noise with an equivalent noise level. When 1% impulsive bad data are introduced, the model still achieves an accuracy of 94.0% and a Macro-F1 score of 93.7%. For the missing-data condition, where 5% of the measurement points are missing and reconstructed by interpolation, the model achieves 94.5% accuracy and 94.2% Macro-F1. These results indicate that the proposed framework can tolerate moderate measurement noise, sparse bad data, and short-term missing measurements.

The robustness of LCGS-Net mainly benefits from its multi-channel temporal encoding and local-contrast/global-smooth graph representation. The temporal encoder extracts compact dynamic features from noisy measurement sequences, while the graph-based dual-branch module exploits both neighborhood contrast and global structural context. As a result, the model does not rely solely on a single noisy measurement channel or an isolated node response, which improves its robustness under imperfect sensing conditions.

For field deployment with actual synchrophasor measurements, several additional adaptations would be required. First, PMU data should be time-aligned and resampled to a consistent sampling rate before being fed into the model. Second, bad-data detection and preprocessing modules are needed to remove gross measurement errors, communication dropouts, and abnormal spikes. Third, online normalization should be applied to reduce the distribution mismatch between simulated training data and field measurements. Finally, model adaptation or fine-tuning using historical PMU records would be necessary to mitigate the domain gap caused by differences between the benchmark simulation model and practical converter-dominated grids. These issues will be further investigated in future work when labeled field oscillation events become available.

### 3.10 Scalability analysis on the IEEE 39-bus system

To further examine the scalability of the proposed LCGS-Net beyond the IEEE 14-bus system, additional validation experiments are conducted on the IEEE 39-bus benchmark system. Compared with the IEEE 14-bus system, the IEEE 39-bus system has a larger network topology, longer electrical propagation paths, and more possible candidate source nodes, which provides a more challenging setting for oscillation source localization.

In this experiment, ten candidate oscillation source buses are selected from Bus 30 to Bus 39. The same data construction procedure, input measurement format, training/testing protocol, and evaluation metrics are adopted as in the IEEE 14-bus case. In addition to Accuracy and Macro-F1, the graph-distance error (GDE) is also reported to quantify the topological deviation between the predicted source bus and the true source bus. A smaller GDE indicates that the predicted source is electrically closer to the actual oscillation source.

As shown in Table 7, all methods show lower performance on the IEEE 39-bus system than on the IEEE 14-bus system, indicating that the larger topology and increased number of candidate sources make the localization task more challenging. Nevertheless, the proposed LCGS-Net still achieves the best performance, with an accuracy of 92.4%, a Macro-F1 score of 92.1%, and the lowest GDE of 0.52. Compared with graph-enhanced baselines such as LSTM-VAE-GCN and Informer-EGAT, LCGS-Net provides higher localization accuracy and smaller graph-distance error. This demonstrates that the proposed local-contrast/global-smooth representation remains effective when the network scale and candidate-source number are increased.

**Table 7:** Scalability validation on the IEEE 39-bus system with ten candidate oscillation sources.

Method	Accuracy (%)	Macro-F1 (%)	GDE
TCN-LSTM	82.6	82.1	1.42
Informer	84.9	84.5	1.27
Autoformer	86.3	86.0	1.16
LSTM-VAE-GCN	88.5	88.2	0.94
Informer-EGAT	90.1	89.8	0.78
**LCGS-Net**	**92.4**	**92.1**	**0.52**

To further analyze the influence of the number of candidate oscillation sources, a sensitivity experiment is conducted by varying *K* on the IEEE 39-bus system. Specifically, three settings are considered: *K* = 5 with candidate buses 30–34, *K* = 7 with candidate buses 30–36, and *K* = 10 with candidate buses 30–39. The results are summarized in Table 8.

**Table 8:** Sensitivity analysis with different numbers of candidate sources on the IEEE 39-bus system.

Number of candidate sources *K*	Candidate buses	Accuracy (%)	Macro-F1 (%)	GDE
5	30–34	95.7	95.5	0.31
7	30–36	94.1	93.8	0.39
10	30–39	92.4	92.1	0.52

As shown in Table 8, the localization performance gradually decreases as the number of candidate sources increases. When *K* increases from 5 to 10, the accuracy decreases from 95.7% to 92.4%, while the GDE increases from 0.31 to 0.52. This trend is reasonable because a larger candidate set introduces more electrically adjacent source nodes and increases the classification difficulty. Even under the most challenging *K* = 10 setting, LCGS-Net still maintains an accuracy above 92%, indicating that the proposed framework has a certain degree of scalability to medium-scale systems.

**Fig 9:**
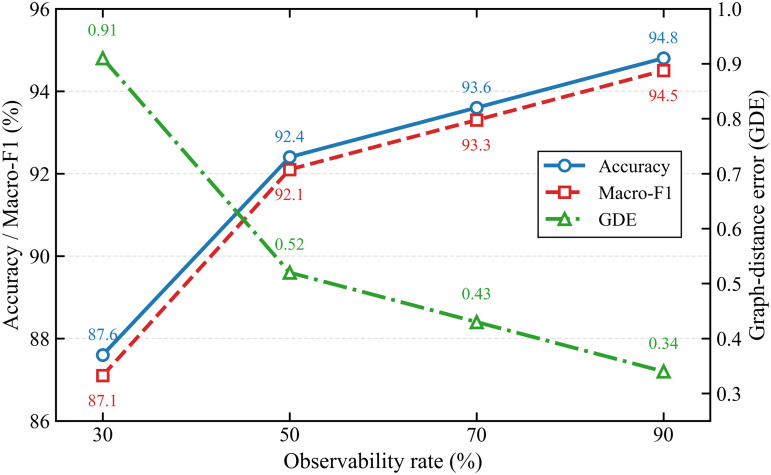
Sensitivity of LCGS-Net to different observability rates on the IEEE 39-bus system.

To further evaluate the influence of partial observability on the IEEE 39-bus system, an additional sensitivity analysis is conducted by varying the bus observability rate under the *K* = 10 setting. Four observability rates are considered, namely 30%, 50%, 70%, and 90%. For each sample, the corresponding proportion of bus measurements is randomly retained, while the remaining bus measurements are masked. The corresponding sensitivity curve is shown in Fig 9.

As shown in Fig 9, the localization performance improves as the observability rate increases. When only 30% of the buses are observed, the accuracy and Macro-F1 are relatively lower, while the GDE is higher, indicating that insufficient spatial measurements increase the ambiguity among electrically adjacent candidate source buses. When the observability rate increases to 50%, the model achieves 92.4% accuracy and 92.1% Macro-F1, which corresponds to the main IEEE 39-bus scalability setting. Further increasing the observability rate to 70% and 90% gradually improves the accuracy and Macro-F1, while reducing the GDE. This trend indicates that richer measurement coverage helps the proposed model capture more complete spatial propagation information. Meanwhile, LCGS-Net still maintains acceptable localization performance under low-observability conditions, demonstrating its robustness to partial measurement availability.

It should be noted that these IEEE 39-bus experiments provide an initial scalability validation rather than a complete demonstration on practical large-scale converter-dominated grids. More extensive evaluations under realistic provincial-grid models, varying line-switching topologies, and larger candidate-source sets will be further investigated in future work. Accordingly, the generalization statements in the abstract and conclusion are revised to avoid overclaiming beyond the current experimental scope.

## 4 Conclusion

This paper presents a novel spatiotemporal graph learning framework, namely LCGS-Net, for wideband oscillation source localization in converter-dominated power systems. Unlike conventional methods that primarily rely on global coherence or low-frequency modal characteristics, the proposed approach explicitly models the dual nature of oscillatory responses by jointly capturing globally smooth propagation patterns and locally contrastive high-frequency perturbations.

To this end, a local-contrast and global-smooth dual-branch representation mechanism is developed to enhance the discriminability of source-related features, while a hierarchical channel interaction module is introduced to exploit the complementary information across multi-channel measurements. In addition, an adaptive fusion strategy enables dynamic balancing between local and global representations under different oscillation conditions.

Simulation results on IEEE benchmark systems show that the proposed method achieves better localization performance than several representative temporal and graph-enhanced baseline models. In particular, LCGS-Net exhibits advantages under high-frequency oscillation scenarios, where localized source-related perturbations are more likely to be weakened by conventional smoothing-oriented graph aggregation. The additional IEEE 39-bus experiments and candidate-source sensitivity analysis provide an initial validation of the scalability of the proposed framework, although they do not fully represent practical large-scale converter-dominated grids. Visualization and ablation studies further indicate that the proposed modules can capture useful local contrastive and global structural features for wideband oscillation localization.

Overall, the proposed framework provides a local-contrast-aware perspective for oscillation source localization. Nevertheless, the present study is still mainly based on benchmark simulation systems, and its applicability to realistic large-scale power grids requires further validation. Future work will focus on larger-scale and more realistic grid models, varying topology conditions, real PMU measurements, multiple simultaneous oscillation sources, and more complex missing-data or asynchronous-sampling scenarios.

It should also be noted that the present study still has limitations when extending LCGS-Net to larger-scale power systems. Although the IEEE 14-bus system provides a controlled benchmark for validating the proposed local-contrast and global-smooth representation mechanism, larger networks may involve more candidate oscillation sources, more complex electrical distances, stronger topology-dependent propagation paths, and higher computational costs. In such cases, the classification difficulty may increase as the number of candidate source nodes grows, and the graph representation may need to be further optimized for scalability and efficiency. Therefore, the current results should be regarded as an initial validation of the proposed framework rather than a complete demonstration on practical large-scale converter-dominated grids. Additional preliminary experiments on the IEEE 39-bus system are conducted to provide an initial scalability check, while more comprehensive validation on larger benchmark systems and real grid models will be considered in future work.

Another limitation of the current formulation is the closed-world assumption of the candidate source set. In this study, the oscillation source localization problem is formulated as a classification task over a predefined candidate set $𝒮=\{s_{1},s_{2},...,s_{K}\}$, where the true source is assumed to be one of the candidate converter buses. If the actual oscillation source originates from a non-candidate bus, such as a non-converter bus or an external disturbance location, the current model will still assign the highest probability to one of the predefined candidate nodes. In this case, the prediction should be interpreted as the most similar or most strongly affected candidate node rather than the true physical source. Therefore, the present framework is mainly applicable to closed-set source localization scenarios. For practical deployment, open-set recognition, confidence-based rejection, or an additional “unknown source” class should be incorporated to identify cases where the true oscillation source is outside the predefined candidate set. This issue will be further investigated in future work.

The current framework also focuses on the localization of a single dominant oscillation source. When multiple oscillation sources occur simultaneously, the measured responses may contain superimposed oscillatory components from different locations, leading to more complex spatial propagation and source-interaction patterns. Under such conditions, the present softmax-based classification head can only output one most probable candidate source, and the prediction may correspond to the dominant source or the candidate node with the strongest observed oscillatory response. Therefore, the current formulation is not directly designed for simultaneous multi-source localization. To handle this situation, the prediction head should be extended from single-label classification to multi-label classification or source-set prediction, for example by replacing the softmax output with independent sigmoid outputs for each candidate node and using a binary cross-entropy loss. In addition, training data containing simultaneous multi-source oscillation events would be required to learn the interaction patterns among different sources. This extension will be considered in future work.

Future work will focus on extending the proposed approach to larger-scale power systems and incorporating more realistic measurement conditions, such as noise, missing data, and asynchronous sampling, to further enhance its applicability in practical scenarios.

## References

[1] Alshahrani S, Khan K, Abido M, Khalid M. Grid-forming converter and stability aspects of renewable-based low-inertia power networks: modern trends and challenges. Arab J Sci Eng. 2023;49(5):6187–216. doi: 10.1007/s13369-023-08399-z

[2] Moore P, Alimi OA, Abu-Siada A. A review of system strength and inertia in renewable-energy-dominated grids: challenges, sustainability, and solutions. Challenges. 2025;16(1):12. doi: 10.3390/challe16010012

[3] Shakerighadi B, Johansson N, Eriksson R, Mitra P, Bolzoni A, Clark A, et al. An overview of stability challenges for power-electronic-dominated power systems: the grid-forming approach. IET Generation Trans & Dist. 2022;17(2):284–306. doi: 10.1049/gtd2.12430

[4] Stanchev P, Hinov N. Smart grids and sustainability in the age of PMSG-dominated renewable energy generation. Energies. 2026;19(3):772. doi: 10.3390/en19030772

[5] Khan M, Wu W, Li L. Grid-forming control for inverter-based resources in power systems: a review on its operation, system stability, and prospective. IET Renewable Power Gen. 2024;18(6):887–907. doi: 10.1049/rpg2.12991

[6] Li C, Huang Y, Deng H, Zhang X, Zhao H. A novel grid-forming technology for transient stability enhancement of power system with high penetration of renewable energy. Int J Electrical Power Energy Systems. 2022;143:108402. doi: 10.1016/j.ijepes.2022.108402

[7] Wang G, Fu L, Hu Q, Liu C, Ma Y. Small-signal synchronization stability of grid-forming converter influenced by multi time-scale control interaction. Energy Rep. 2023;9:597–606. doi: 10.1016/j.egyr.2022.11.137

[8] Zheng L, Liu X. Multi-timescale energy-aware grid-forming control with self-tuning virtual inductance for battery lifetime enhancement. Sci Rep. 2026;16(1):15926. doi: 10.1038/s41598-026-47270-7 41933022 PMC13195112

[9] Liu N, Wang H, Zhou D, Shi H, Chen Z. Comprehensive review of power system oscillations in large-scale power electronic-based renewable energy power plants. J Renew Sustain Energy. 2023;15(4). doi: 10.1063/5.0148188

[10] Nazar M, Abbas S, Asghar S, Saleem S, Abutuqayqah H, AL Garalleh H, et al. Comparison of unsteady MHD flow of second grade fluid by two fractional derivatives. Numerical Heat Transfer, Part A: Applications. 2024;87(1). doi: 10.1080/10407782.2024.2374536

[11] Rafique U, Nazar M, Abbas S, Elnaggar GR, Arishi A, Abdullaeva B. Investigation of a novel thermal and mass stratification effect on second grade fluid: applications in industrial heat transfer systems. Case Studies in Thermal Eng. 2025;73:106399. doi: 10.1016/j.csite.2025.106399

[12] Abbas S, Gilani SFF, Nazar M, Fatima M, Ahmad M, Nisa ZU. Bio-convection flow of fractionalized second grade fluid through a vertical channel with Fourier’s and Fick’s laws. Mod Phys Lett B. 2023;37(23). doi: 10.1142/s0217984923500690

[13] Abbas S, Ramzan M, Inam I, Saleem S, Nazar M, Abduvalieva D, et al. Analysis of fractionalized Brinkman flow in the presence of diffusion effect. Sci Rep. 2024;14(1):22507. doi: 10.1038/s41598-024-72785-2 39341809 PMC11439061

[14] Ramzan M, Shafique A, Abbas S, Ali R, Lamoudan T, Jan R, et al. Mathematical analysis of Prandtl number with Artificial neural network and fractional operator for nanofluid flow. Case Studies in Thermal Engineering. 2025;71:106164. doi: 10.1016/j.csite.2025.106164

[15] Khazaei J, Zhao Y, Huang Z, et al. Locating sources of forced oscillations in power systems using PMU data. IEEE Transac Power Systems. 2018.

[16] Amin M, Molinas M. Small-signal stability assessment of power electronics based power systems: a discussion of impedance- and eigenvalue-based methods. IEEE Trans on Ind Applicat. 2017;53(5):5014–30. doi: 10.1109/tia.2017.2712692

[17] Yao W, Jiang L, Wen J, Wu QH, Cheng S. Wide-area damping controller of FACTS devices for inter-area oscillations considering communication time delays. IEEE Trans Power Syst. 2014;29(1):318–29. doi: 10.1109/tpwrs.2013.2280216

[18] Maslennikov S, Wang B, Litvinov E. Dissipating energy flow method for locating the source of sustained oscillations. Inter J Elect Power Energy Systems. 2017;88:55–62. doi: 10.1016/j.ijepes.2016.12.010

[19] An S, Qiu W, Pu Q, Chen S, Zheng Y, Duan J, et al. Power system wideband oscillation estimation, localization, and mitigation. IET Generation Trans Dist. 2023;17(11):2655–66. doi: 10.1049/gtd2.12845

[20] Chen Y, Fan Z, Gregory D, Zhou X, Rabbani R. A survey of oscillation localization techniques in power systems. IEEE Access. 2025;13:28836–60. doi: 10.1109/access.2025.3540318

[21] Li L, Wang B, Liu S, Li J, Teng S, Liu D, et al. Forced oscillation location based on temporal graph convolutional network. Energy Reports. 2023;9:646–54. doi: 10.1016/j.egyr.2023.03.013

[22] Li C, Wang Y, Zheng Z. A broadband oscillation source location method based on LSTM variational autoencoder and graph convolutional neural network. IET Conf Proc. 2023;2023(27):87–93. doi: 10.1049/icp.2023.3032

[23] Li C, Wang Y, Zheng Z. A broadband oscillation source location method based on LSTM variational autoencoder and graph convolutional neural network. In: Proceedings of the IEEE Conference; 2023.

[24] Hu X, Yang J, Gao Y, Zhang Z, Jin S, Wang C. New power system wide-frequency oscillation source localization method based on informer-EGAT multimodal spatio-temporal collaborative perception. In: 2025 8th International conference on energy, electrical and power engineering (CEEPE), 2025. 185–90. 10.1109/ceepe64987.2025.11033925

[25] Cui Z, Hu W, Zhang G, Huang Q, Chen Z, Blaabjerg F. Knowledge-informed deep learning method for multiple oscillation sources localization. IEEE Trans Power Syst. 2025;40(3):2811–4. doi: 10.1109/tpwrs.2025.3533968

[26] Chen L, Li H, Xie X, Liu Y, Lin Y. A review of centralized control technologies and prospects of distributed control technologies for wideband oscillations. Renew Sustain Energy Rev. 2026;232:116840. doi: 10.1016/j.rser.2026.116840

[27] Qiu L, Gu M, Chen Z, Du Z, Zhang L, Li W, et al. Oscillation suppression of grid-following converters by grid-forming converters with adaptive droop control. Energies. 2024;17(20):5230. doi: 10.3390/en17205230

[28] Liu Z, Li D, Wang W, Wang J, Gong D. A review of the research on the wide-band oscillation analysis and suppression of renewable energy grid-connected systems. Energies. 2024;17(8):1809. doi: 10.3390/en17081809

[29] Xie X, Shair J. Oscillatory stability of converter-dominated power systems. Cham: Springer; 2024.doi: 10.1007/978-3-031-53357-0

[30] An S, Qiu W, Pu Q, Chen S, Zheng Y, Duan J, et al. Power system wideband oscillation estimation, localization, and mitigation. IET Generation Trans & Dist. 2023;17(11):2655–66. doi: 10.1049/gtd2.12845

[31] Li T, Ledwich G, Mishra Y, Chow JH, Vahidnia A. Wave aspect of power system transient stability—part i: finite approximation. IEEE Trans Power Syst. 2017;32(4):2493–500. doi: 10.1109/tpwrs.2016.2621006

[32] Olmon RL, Raschke MB. Antenna-load interactions at optical frequencies: impedance matching to quantum systems. Nanotechnology. 2012;23(44):444001. doi: 10.1088/0957-4484/23/44/444001 23079849

[33] Hammond DK, Vandergheynst P, Gribonval R. Wavelets on graphs via spectral graph theory. Appl Comput Harmonic Analysis. 2011;30(2):129–50. doi: 10.1016/j.acha.2010.04.005

[34] Defferrard M, Bresson X, Vandergheynst P. Convolutional neural networks on graphs with fast localized spectral filtering. In: Advances in neural information processing systems.29; 2016. 3844–52.

[35] He M, Wei Z, Xu H. BernNet: learning arbitrary graph spectral filters via bernstein approximation. In: Advances in neural information processing systems. 34; 2021.14239–51.

[36] Derr T, Ma Y, Tang J. Signed graph convolutional network. In: 2018 IEEE International Conference on Data Mining; 2018. 929–34.doi: 10.1109/ICDM.2018.00113

[37] Zhu J, Yan Y, Zhao L, Heimann M, Akoglu L, Koutra D. Beyond homophily in graph neural networks: current limitations and effective designs. In: Advances in neural information processing systems. 33; 2020. 7793–804.

[38] Bai S, Kolter JZ, Koltun V. An empirical evaluation of generic convolutional and recurrent networks for sequence modeling. arXiv preprint arXiv:180301271. 2018. doi: 10.48550/arXiv.1803.01271

[39] Zhou H, Zhang S, Peng J, Zhang S, Li J, Xiong H, et al. Informer: beyond efficient transformer for long sequence time-series forecasting. AAAI. 2021;35(12):11106–15. doi: 10.1609/aaai.v35i12.17325

[40] Wu H, Xu J, Wang J, Long M. Autoformer: decomposition transformers with auto-correlation for long-term series forecasting. In: Advances in neural information processing systems (NeurIPS). 34; 2021. 22419–30.

